# Improving the potential of ethyl acetate green anti-solvent to fabricate efficient and stable perovskite solar cells

**DOI:** 10.1039/d2ra05454j

**Published:** 2022-11-14

**Authors:** Mustafa K. A. Mohammed, Sangeeta Singh, Ali K. Al-Mousoi, Rahul Pandey, Jaya Madan, Davoud Dastan, G. Ravi

**Affiliations:** Radiological Techniques Department, Al-Mustaqbal University College 51001 Hillah Babylon Iraq mustafa_kareem97@yahoo.com; Microelectronics Lab, National Institute of Technology Patna 800005 India; Department of Radiology and Ultrasonography Techniques, College of Medical Techniques, Al-Farahidi University Baghdad Iraq; VLSI Centre of Excellence, Chitkara University Institute of Engineering and Technology, Chitkara University 140417 Punjab India; Department of Materials Science and Engineering, Cornell University Ithaca NY 14850 USA; Department of Physics, Alagappa University Karaikudi 630003 Tamil Nadu India

## Abstract

Until now, in all state-of-the-art efficient perovskite solar cells (PSCs), during the fabrication process of the perovskite layer, highly toxic anti-solvents such as toluene, chlorobenzene, and diethyl ether have been used. This is highly concerning and urgently needs to be considered by laboratories and institutes to protect the health of researchers and employees working towards safe PSC fabrication. Green anti-solvents are usually used along with low-performance PSCs. The current study solves the ineptitude of the typical ethyl acetate green anti-solvent by adding a potassium thiocyanate (KSCN) material to it. The KSCN additive causes delay in the perovskite growing process. It guarantees the formation of larger perovskite domains during fabrication. The enlarged perovskite domains reduce the bulk and surface trap density in the perovskite. It enables lower trap-facilitated charge recombination along with efficient charge extraction in PSCs. Overall, the developed method results in a champion performance of 17.12% for PSCs, higher than the 13.78% recorded for control PSCs. The enlarged perovskite domains warrant lower humidity interaction paths with the perovskite composition, indicating higher stability in PSCs.

## Abstract 2

The current study solves the ineptitude of the typical ethyl acetate green anti-solvent by adding potassium thiocyanate (KSCN).

## Graphical abstract

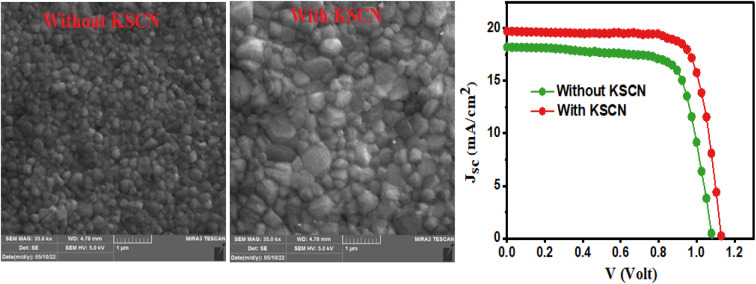

## 1. Introduction

Perovskites are one of the most promising semiconductors for light-harvesting missions in the new generation of photovoltaics.^1–4^ Researchers have developed various methods for fabricating perovskite solar cells (PSCs), such as one-step,^5^ two-step,^6,7^ anti-solvent assisted,^8^ sequential deposition,^9^ vapor-assisted solution process,^10^ and chemical vapor deposition,^11^*etc.* To date, all reported state-of-the-art efficient PSCs have been fabricated with the anti-solvent assisted method.^12–15^

In the anti-solvent assisted method, a specific volume of one of the solvents such as chlorobenzene (CB), toluene, ethylene glycol, 1,2-dichlorobenzene, diethyl ether, and chloroform is immediately or slowly dripped on the intermediate perovskite phase to extract host solvents, resulting in a fast deposition-crystallization along with an instantaneous color change during the spin-coating procedure.^16–20^ The first drawback of the anti-solvent approach is the use of predominantly toxic solvents that are dangerous to the researcher. To tackle the toxicity of anti-solvents, green solvents such as anisole,^21^ ethyl acetate,^22^ and methoxybenzene^23^ have been introduced. The randomly oriented and distributed perovskite crystals formed are another disadvantage of the anti-solvent, which reduces the PSC performance along with an undesirable stability behavior. In particular, pouring an antisolvent on the intermediate perovskite layer produces many nuclei with an immediate decrease in solubility, which is not a controllable process.^24,25^

To find ways to control the crystallization process in the anti-solvent assisted method, the phrase “anti-solvent engineering” has emerged. The mixing of anti-solvents is a method to modulate the formation process of perovskite. Yu *et al.* introduced a hybrid anti-solvent using ethyl ether and *n*-hexane solvents.^26^ They showed that ethyl ether is miscible with *N*,*N*-dimethylformamide (DMF), promoting the nucleation process in the whole precursor solution. In contrast, the impact of *n*-hexane is restricted because of its immiscibility with DMF. From this view, they concluded that *n*-hexane-rich mixed anti-solvents have a lower nucleation density during perovskite formation than ethyl ether-rich mixed anti-solvents. Controlling the temperature of an anti-solvent is another way to optimize its potential to form a dense and favorable perovskite layer for photovoltaic applications.^25^ Another method for tailoring their roles during perovskite layer formation is to incorporate materials or solutions into the anti-solvent. Li *et al.*^27^ introduced 6% acetonitrile into the CB anti-solvent and obtained a power conversion efficiency (PCE) of 18.9% for methylammonium lead iodide (MAPbI_3_)-based devices. They concluded that the acetonitrile reacts with excess dimethyl sulfoxide (DMSO) in the intermediate phase of the MAPbI_3_ film and leads to larger grains in the perovskite layer. Gao *et al.*^28^ used cesium lead bromide (CsPbBr_3_) nanoparticles as an additive for the CB anti-solvent. They observed that nanoparticles operate as nucleation sites to form uniform and smooth films. The CsPbBr_3_ additive acts as a passivator for the MAPbI_3_ layer to enlarge the perovskite grains and suppresses charge recombination in the perovskite layer. Albeit heavy attempts made by researchers to improve the role of anti-solvents in perovskite formation, usually their anti-solvents are toxic, and there is a lack of anti-solvent engineering for green anti-solvents.

In the current study, we planned to improve the potential of ethyl acetate green anti-solvent (GAS). We employed potassium thiocyanate (KSCN) as an additive source for GAS. Volatile thiocyanate ions (SCN^−^) such as ammonium thiocyanate,^29^ lead(ii) thiocyanate,^30^ guanidinium thiocyanate,^31^ or methylammonium thiocyanate^32^ have been widely used in PSCs to order the lead–iodide (Pb–I) octahedrons through the strong affinity between Pb^2+^ and SCN^−^ and optimize the film morphology. In addition, K^+^ alkali metal ions could effectively passivate the perovskite grain boundaries and trap states, increasing the stability and efficiency of solar cells.^33^ A series of tests were conducted to monitor the effects of KSCN on perovskite formation and then to find reasons for photovoltaic improvements in the corresponding PSCs. The results show that KSCN reduces the activation energy of MAPbI_3_ crystallization and enlarges the perovskite domain sizes together with passivated domain boundaries. The results demonstrated that the higher efficiency of PSCs obtained with the help of the KSCN additive are due to lower trap surfaces and charge recombination sites. The KSCN-tailored PSCs show more favourable long-term stability behaviour than those of the control PSC devices. It can be concluded that the anti-solvent engineering for GAS is a promising route to designing a more environmentally friendly fabrication process for PSCs.

## 2. Experimental part

### 2.1 Solution synthesis

645.4 mg of lead iodide (PbI_2_, Lumtec, 99.8%) was dissolved in 1 mL of DMF (Merck, 99.8%) and DMSO (Merck, 99.8%) in a volume ratio of 9 : 1 to obtain a 1.4 M PbI_2_ solution. The resultant PbI_2_ was mixed at 80 °C for 30 min. 222.6 mg of methylammonium iodide (MAI, 99%) was added to a pre-solution of PbI_2_ and shaken with hand for 5 min to synthesize MAPbI_3_. Then, 100 mg of TiO_2_ paste (SunLab, 20 NR paste) is dispersed in 600 mg of ethanol (EtOH, Merck, 99.7%) and mixed at RT for 12 h to prepare a homogeneous mesoscopic TiO_2_ (p-TiO_2_) solution. Our compact TiO_2_ precursor was prepared as in our previous research.^34^

### 2.2 Device fabrication

Transparent fluorine-doped tin oxide (FTO) glasses were ultrasonically patterned with deionized water, EtOH, propanol, and isopropanol (IPA), each step for 20 min. 50 μL of the c-TiO_2_ pre-solution is poured over FTO glasses and spun at 4000 rpm for 35 s, followed by sintering at 500 °C for 45 min to prepare a c-TiO_2_ thin film. 75 μL of p-TiO_2_ pre-solution is deposited over each c-TiO_2_ thin-film at 4000 rpm for 35 s, followed by sintering at 500 °C for 45 min. Perovskite films were prepared by pouring 75 μL of MAPbI_3_ precursor over each substrate, followed by depositing at 1000 rpm for 10 s and 5000 for 45 s. During the faster step, 120 μL of pure ethyl acetate or KSCN-containing ethyl acetate was dynamically poured on the perovskite layers to assist their crystal growth. After that, the perovskites were heated for 40 min at 98 °C. Copper(ii) phthalocyanine (CuPc, 99%, Aldrich) material was utilized as a hole transport layer (HTL). A 30 nm CuPc was deposited on the MAPbI_3_ film by thermal evaporation.^35^ Finally, a 90 nm gold layer was evaporated on the MAPbI_3_ layers.

### 2.3 Characterization

Scanning electron microscope (FE-SEM) instrument model of Mira3, TESCAN was employed to investigate MAPbI_3_ layers' top morphology. An X-ray diffractometer (XRD) instrument (Bruker-D8 model) recorded the XRD patterns of MAPbI_3_ films. A LAMBDA 1050 ultraviolet-visible (UV-vis) spectrometer investigated the absorption ability of MAPbI_3_ layers. A HORIBA Fluorolog-III photospectrometer measured the photoluminescence (PL) spectrum of MAPbI_3_ layers.^36^ The current–voltage (*J*–*V*) performance of MAPbI_3_ devices with a masked area of 2 mm × 4 mm was measured using a Keithley 2401 under a simulated 100 mW cm^−2^ illumination.

## 3. Results and discussion

**Fig. 1:**
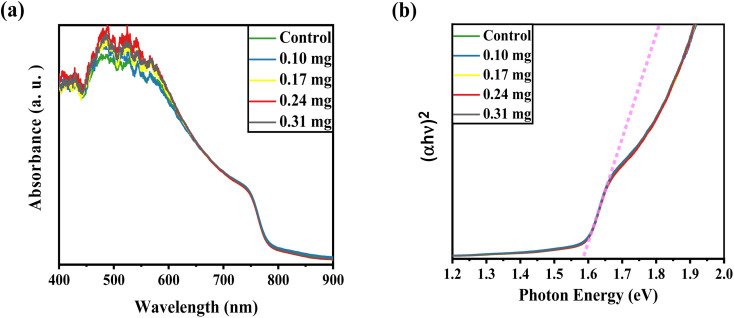
(a) UV-vis patterns and (b) related Tauc plots of the perovskite films fabricated on the FTO/c-TiO_2_/mp-TiO_2_ electrodes by employing anti-solvents, *i.e.*, anti-solvent with various amounts of potassium thiocyanate (0.1 mg, 0.17 mg, 0.24 mg, and 0.31 mg).

UV-vis absorption was conducted to record the optical characteristics of the perovskite layers before and after KSCN inclusion. Fig. 1a demonstrates similar absorbance trends for films with and without KSCN modification. Furthermore, it is obvious that the perovskite with KSCN inclusion, particularly 0.24 mg KSCN, absorbs more illumination than the untreated film, which might be attributed to the improvement of morphology and crystallinity.^37^ As illustrated in Fig. 1b, the optical bandgap of the perovskite layer in the absence of KSCN is approximately 1.582 eV, in line with earlier reports.^15,38^ There is no noticeable change in the bandgap of perovskites after treating them with various concentrations of KSCN.

**Fig. 2:**
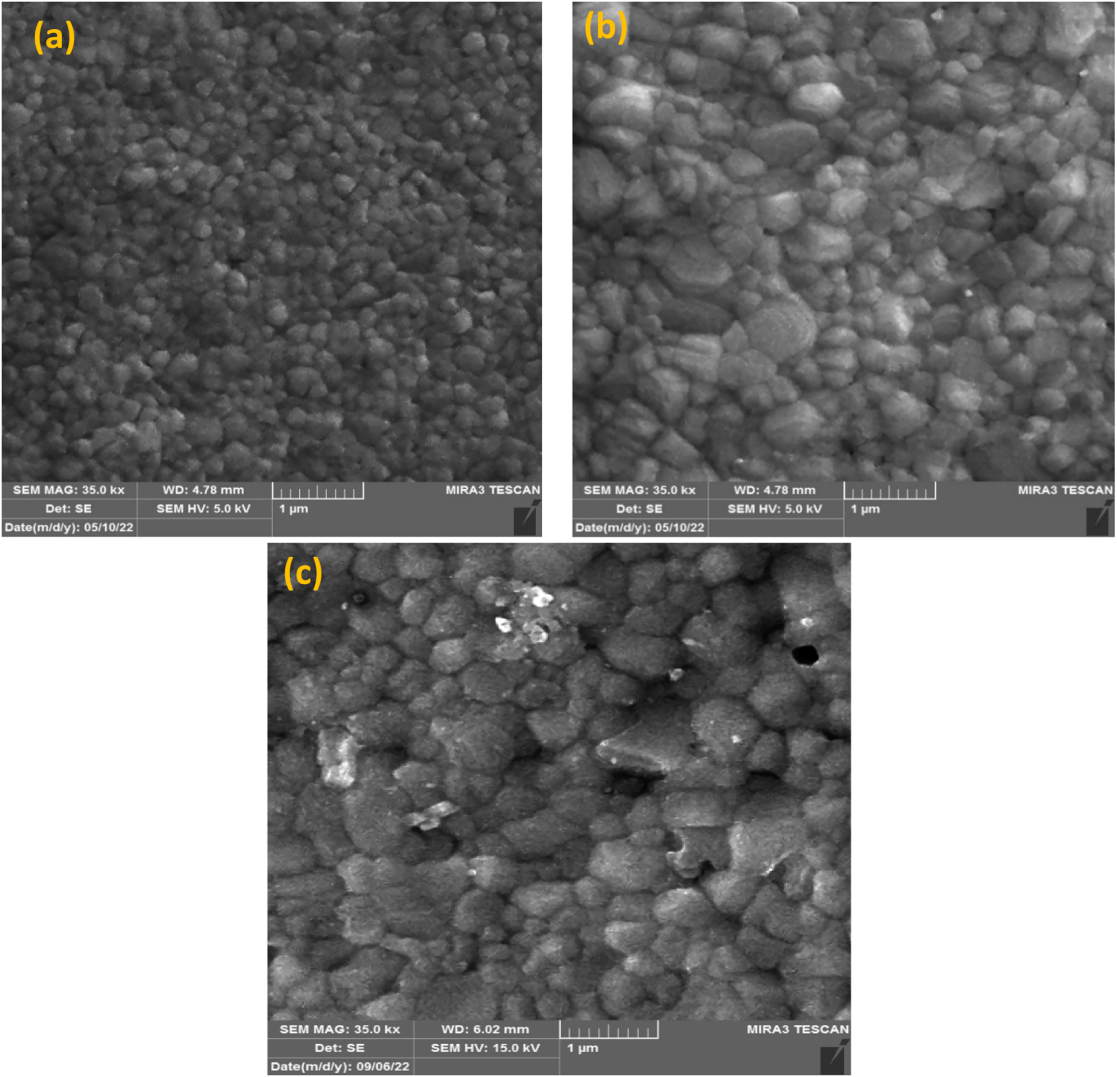
FESEM images of perovskites fabricated with (a) pure and (b) 0.24 mg and (c) 0.31 mg of potassium thiocyanate-containing ethyl acetate anti-solvents.

We used FESEM measurements to confirm the impact of KSCN on the quality of the MAPbI_3_ film. The FESEM images, as presented in Fig. 2, indicate the distribution of grain size and the quality of perovskites without and with 0.24 mg KSCN additive. Both films exhibit normal perovskite crystalline characteristics. Fig. 2a shows that the perovskite without treatment has a smaller grain size of roughly 150 nm and a large number of grain domains. The KSCN-treated perovskite attains a pinhole-free and dense film with an actual enhancement in grain size of about 350 nm with the inclusion of 0.24 mg KSCN (Fig. 2b). As a result, the pinholes, small grains, and grain domains that might potentially cause flaws and recombination within the perovskite would be significantly minimized after KSCN is added.^31^ As can be seen in Fig. 2c, grain size remains still large, but some surface defects exist in this layer, concluding that an excess amount of KSCN leads to destroying perovskite crystallinity.

**Fig. 3:**
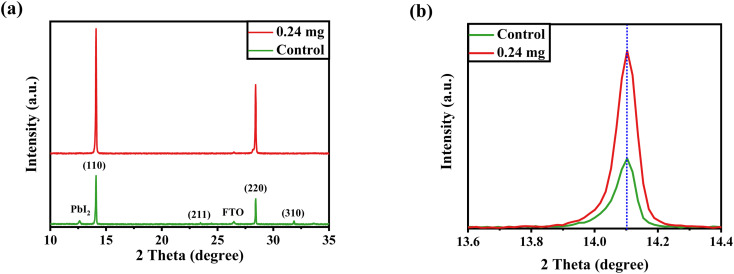
(a) XRD profiles of perovskite layers fabricated on FTO with the assistance of pure anti-solvent or KSCN-containing anti-solvent. (b) Zoomed XRD of perovskites around 2*θ* = 14°.

After confirming the morphological and optical properties of perovskite, we performed XRD to affirm the crystal phase and investigate the crystallization of the perovskite layer after being treated with 0.24 mg of KSCN additive. It has been demonstrated that, in addition to improving perovskite morphology, KSCN can enhance the perovskite crystalline phase. Both samples, as displayed in Fig. 3a, exhibit prominent peaks in intensity at 14.12° and 28.45°, which can be indexed to the (110) and (220) facets of MAPbI_3_, indicating the fabrication of a well-oriented tetragonal MAPbI_3_ structure.^39^ Other than the PbI_2_ peak, no other crystalline defects were recognized, proving the MAPbI_3_ crystal's phase purity. Besides, the intensity of the dominant (110) peak is improved with the incorporation of KSCN (Fig. 3b), implying increased crystallinity for MAPbI_3_ crystal was achieved compared with the film fabricated without KSCN. Due to the partial degradation of the perovskite to PbI_2_, the diffraction pattern at 12.3° could be ascribed to cubic PbI_2_. Surplus PbI_2_ in the MAPbI_3_ layer would be undesirable for charge transportation, decreasing film stability and increasing the hysteretic effect of PSCs.^17^ Simultaneously, the typical peak intensity of PbI_2_ was significantly suppressed with the loading of KSCN.

The FE-SEM and XRD findings implied that the crystalline structure and morphology of MAPbI_3_ crystals could be enhanced by the addition of KSCN. As reported in the literature, because SCN^−^ is a strong coordination bond linking group, the coupling between Pb^2+^ and SCN^−^ is substantially greater than that between Pb^2+^ and I^−^. Moreover, the coordination of SCN^−^ may decrease the activation energy of crystals as well as the interaction of Pb^2+^ with DMSO. As a result, the addition of SCN coupled with Pb^2+^ may delay MAPbI_3_ crystal nucleation and lead to the formation of large-sized crystals. Hence, the MAPbI_3_ film's grain size was enlarged.^40–42^

**Fig. 4:**
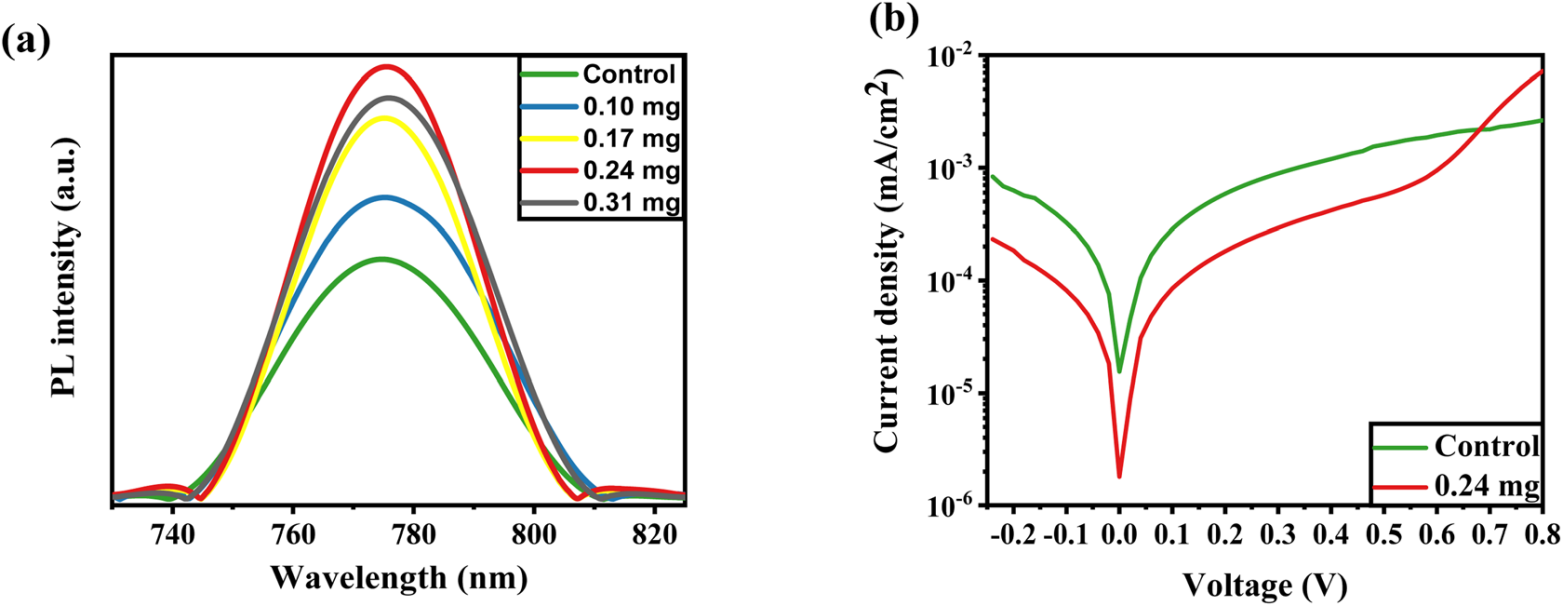
(a) PL spectrum of perovskite layers fabricated with pure or potassium thiocyanate (KSCN)-containing ethyl acetate anti-solvents on the glass. (b) Dark *J*–*V* curves PSCs with pure and KSCN-containing ethyl acetate anti-solvents.

Fig. 4a illustrates the PL spectra for perovskite fabricated with the assistance of KSCN at different concentrations. The PL profile achieves its maximum peak at a concentration of 0.24 mg, but the peak weakens when the concentration reaches 0.31 mg. This could imply higher radiative recombination during KSCN modification, resulting in improved crystallinity, elimination of grain domains, and reduction of pinholes. However, a 0.31 mg KSCN inclusion, for example, could destroy the crystalline structure of MAPbI_3_, resulting in a decrease in PL intensity.^40,43^ Also, the maximum intensity is found at the KSCN concentration of 0.24 mg, which displays the lowest trap density in the perovskite. The decrease in intrinsic defects leads to an increase in carrier lifetime in MAPbI_3_.^44^

The dark *J*–*V* plots of devices based on control and KSCN-treated perovskites are exhibited in Fig. 4b. As revealed, the cell with KSCN exhibits a lower leakage current in comparison to that of the untreated cell, further indicating the enhanced photocarrier transport and reduced charge recombination by KSCN.^45^ The PSC with untreated perovskite has a reverse saturation *J*_o_ of 1.5 × 10^−5^ mA cm^−2^. In the case of 0.24 mg KSCN PSC, the *J*_o_ value is remarkably suppressed to 1.7 × 10^−6^ mA cm^−2^, implying a decreased number of shallow traps with KSCN treatment.^46^

**Fig. 5:**
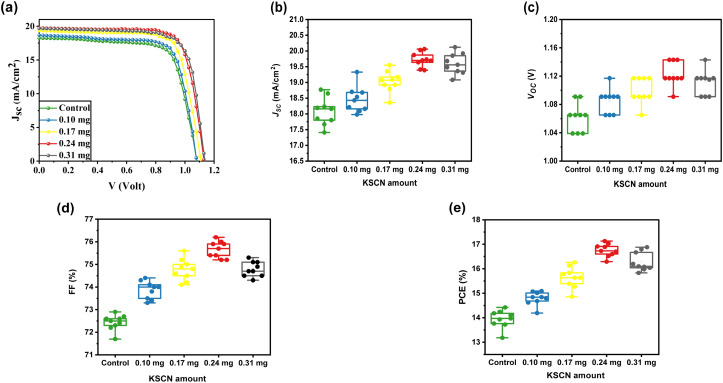
(a) *J*–*V* curves of the champion PSCs constructed with different GASs. Statistical analysis of PV parameters including (b) *J*_SC_, (c) *V*_OC_, (d) FF, and (e) PCE. The data were collected from 10 individual KSCN-engineered anti-solvent PSCs.

The MAPbI_3_-based solar cells were constructed in order to study the impact of KSCN modifications on PV merits under the typical AM 1.5 simulator. Fig. 5a depicts the measured *J*–*V* profiles of PSCs based on MAPbI_3_ layers with varying contents of KSCN additives, and Table 1 summarizes the relative PV parameters. The PSC developed without utilizing KSCN GAS treatment under the reverse scan yielded a PCE of 14.42%, *J*_SC_ of 18.23 mA cm^−2^, *V*_OC_ of 1.091 V, and FF of 72.50%. The PSC performance results were all improved when the perovskite film was treated with KSCN-containing anti-solvents. The optimum cell performance was obtained with a performance of 17.13%, *J*_SC_ of 19.73 mA cm^−2^, *V*_OC_ of 1.143 V, and an FF of 75.90% utilizing 0.24 mg of KSCN additive. Nevertheless, when the KSCN amount was increased to 0.31 mg, the *J*_SC_, FF, and PCE of the cell decreased rather than increased. We analyzed the PV parameters for ten individual devices based on different KSCN treatments to assess the reliability and reproducibility of devices with remarkable performance. As exhibited in Fig. 5b–e, good repeatability and performance of devices were accomplished by the utilization of the KSCN-contained GAS due to the high-quality perovskite compared to the control device.

**Table 1:** *J*–*V* parameters of MAPbI_3_-based devices fabricated with different anti-solvents

Sample		*V*_OC_ a (V)	*J*_SC_ b (mA cm^−2^)	FF^c^ (%)	PCE (%)
Control	Average	1.062	18.08	72.43	13.94
	Best	1.091	18.23	72.50	14.42
0.10 mg	Average	1.085	18.46	73.87	14.79
	Best	18.68	18.68	74.00	15.09
0.17 mg	Average	1.010	19.02	74.78	15.64
	Best	1.117	19.35	75.20	16.26
0.24 mg	Average	1.123	19.75	75.66	16.76
	Best	1.143	19.73	75.90	17.13
0.31 mg	Average	1.111	19.60	74.79	16.29
	Best	1.143	19.68	75.10	16.88

a *V*_OC_ is open-circuit voltage.

b *J*_SC_ is short-circuit current density.

c FF is fill factor.

**Fig. 6:**
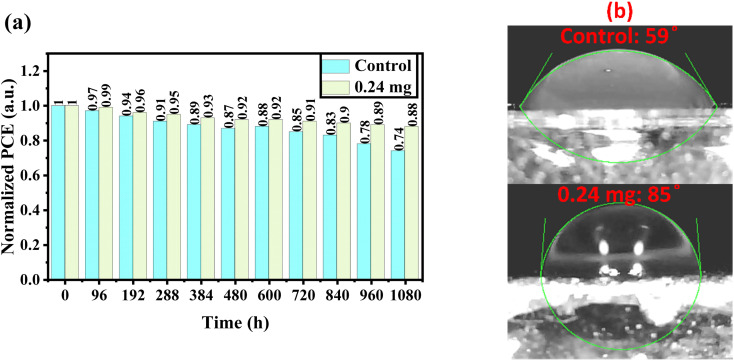
(a) Stability measurements of PSCs with pure or potassium thiocyanate (KSCN)-containing anti-solvents stored in air with RH of 20–30%. (b) Contact angle test of water droplets on the perovskite layers fabricated with pure or KSCN-contained anti-solvents.

The long-term stability of perovskite devices based on the control and KSCN-treated perovskites was traced as a function of storage time under air at a relative humidity (RH) of 20–30%. From Fig. 6a, when the control devices without any sealing were exposed to the environment, the efficiency showed low decomposition after 8 days (6%). But, when the device was exposed to the same conditions for a long period, efficiency decreased quickly to 74% of its initial performance. Interestingly, the KSCN-based device outperforms the control MAPbI_3_ in terms of long-term stability. Even after 45 days, the KSCN-based device exhibits a 12% degradation, indicating acceptable performance stability. Obviously, the inorganic HTL-based PSC is stable when it is treated with the KSCN-based GAS method, making the perovskite of interest for utilization in practical optoelectronic applications. Water contact angle experiments were carried out to thoroughly explore the susceptibility of the MAPbI_3_ perovskite to humidity. It could be concluded that KSCN-based GAS passivates perovskite grain boundaries, blocking humidity diffusion to the perovskite layer through them and keeping perovskite safe from humidity degradation.^47,48^ In other words, the hydrophobicity of the MAPbI_3_ layer improves following the addition of KSCN-processed GAS. Fig. 6b shows the contact angles of water drops on the surface of MAPbI_3_ films, which are 59° and 85°, respectively. The enhanced wettability suggests increased humidity tolerance in the atmosphere, resulting in slower perovskite degradation, indicating that the described KSCN-assisted GAS spin-coating can successfully stabilize the MAPbI_3_ structure and mitigate moisture deterioration.^49^

## 4. Conclusion

In summary, we reported how to enhance efficiency and long-term stability in standard (n–i–p) perovskite solar cells based on the inorganic CuPc hole-transport layer. Potassium thiocyanate was added to the eco-friendly ethyl acetate anti-solvent to assist the one-step spin-coating deposition of the MAPbI_3_ perovskite film. The incorporated KSCN additive acts synergistically to enhance the grain size and crystallinity of the perovskite. When 0.24 mg of KSCN is added, the grain size increases to over 350 nm, which is more than three times that of untreated perovskite. The observed PL spectra and dark *J*–*V* show that the perovskite film with KSCN had decreased trap states and enhanced charge transfer. The devices attained a PCE of 17.13% with improved ambient stability at the optimal concentration of the KSCN additive. The high-quality MAPbI_3_ perovskite that we developed with large grain size, increased crystallinity, fewer grain boundaries, and suppressed charge recombination appears to be responsible for PV performance. As a result of these findings, including KSCN additive into GAS is a simple and promising strategy for increasing the PV performance and stability of MAPbI_3_ photovoltaics.

## Data availability

Data will be available based on reasonable request.

## Conflicts of interest

The authors declare no conflict of interest.

## Supplementary Material
